# High-Precision Absolute Pose Sensing for Parallel Mechanisms

**DOI:** 10.3390/s22051995

**Published:** 2022-03-03

**Authors:** Constantin Schempp, Stefan Schulz

**Affiliations:** 1 Physik Instrumente (PI) GmbH & Co. KG, Auf der Roemerstraße 1, 76228 Karlsruhe, Germany; c.schempp@pi.de; 2 Faculty of Mechanical Engineering and Mechatronics, Karlsruhe University of Applied Sciences, Moltkestraße 30, 76133 Karlsruhe, Germany

**Keywords:** parallel kinematics, machine vision, camera system, fiducial tags, direct kinematic problem, high precision

## Abstract

A parallel mechanism’s pose is usually obtained indirectly from the active joints’ coordinates by solving the direct kinematics problem. Its accuracy mainly depends on the accuracy of the measured active joints’ coordinates, the tolerances in the active and passive joints, possible backlash, axes misalignment, limb deformations due to stress or temperature, the initial pose estimate that is used for the numerical method, and the accuracy of the kinematic model itself. Backlash and temperature deformations in the active joints especially hinder high-precision applications as they usually cannot be observed. By implementing a camera module on the base platform and an array of fiducial tags on the moveable manipulator platform of a parallel mechanism, a highly accurate, direct, and absolute pose measurement system can be obtained that can overcome those limitations. In this paper, such a measurement system is proposed, designed, and its accuracy is investigated on a state-of-the-art H-811.I2 6-axis miniature hexapod by Physik Instrumente (PI) GmbH & Co. KG.

## 1. Introduction

Parallel mechanisms are famous for their higher position accuracy compared to serial mechanisms due to the higher stiffness and the parallel arrangement of their limbs that connect the fixed base platform with the moveable manipulator platform. They furthermore offer lower moving masses, smaller overall machine sizes, lower inertial forces, and faster processing velocities. While errors in serial mechanisms, such as backlash or axes misalignment and limb deformations, can add up to huge position errors of the manipulator platform, errors in parallel mechanisms only lead to comparably small position errors [1,2,3,4,5].

The Stewart–Gough platform is especially widely used for high-precision applications due to its parallel kinematic structure, six degrees of freedom, and simple kinematic architecture. The Stewart–Gough platform, named after its inventors Gough [6] and Stewart [7], has three translational and three rotational degrees of freedom and consists of six identical limbs that connect the fixed base platform with the moveable manipulator platform. One limb itself consists of a length-variable linear actuator (P-joint) and joints on both ends, i.e., a spherical joint (S-joint), with three degrees of freedom on the one end and a universal joint (U-joint) with two degrees of freedom on the other end. Consequently, the Stewart–Gough platform is called a 6-UPS parallel mechanism. The underlined joint indicates that this joint is active and actuated while the other joints are passive and align according to the closure constraints of the mechanism.

Parallel mechanisms also have a number of known limitations, including restricted workspace mobility, singularities, smaller workspace to size ratio, and higher complexity of control [5,8,9,10]. The effect of error compensation that ideally leads to the above-mentioned higher position accuracy is brought with the effect of larger reaction forces in the passive joints. The difficulty in control mainly emanates from the direct kinematics problem, which arises from the fact that the active joints’ coordinates, i.e., the lengths of the linear actuators, are not sufficient for unambiguously determining the pose of the manipulator platform. In fact, a clear assignment between the active joints’ coordinates and the manipulator platform’s pose is not possible [11,12,13,14,15]. By using a iterative methods such as the Newton–Raphson procedure and a pose estimate or evolutionary algorithms, the actual pose of the manipulator platform can be found [16,17,18,19,20,21,22]. As the pose of a parallel mechanism is not measured directly but instead solely estimated from the measured active joints’ coordinates (for the Stewart–Gough platform from the lengths of the linear actuators), its accuracy is vulnerable to several failure sources. These include, for example, tolerances in the active and passive joints, possible backlash, axes offsets or misalignment, and limb deformations due to stress or temperature [9,23]. In the following, these limitations are reviewed.

As mentioned above, the parallel mechanism’s pose cannot be calculated unambiguously and is usually computed with the Newton–Raphson algorithm and a pose estimate. The initial pose estimate and the accuracy of the kinematic model itself directly influence the accuracy of the determined pose [5,24]. If, for example, the inverse kinematics equations are not 100% correct, the pose obtained from the active joints’ coordinates does not match with the real pose. This can happen if the joints’ positions are uncertain (e.g., due to manufacturing tolerances) or the universal joints’ axes do not intersect in a common point.

Furthermore, possible mechanical clearances in the drive train, for example, in the gear heads, bearings, or joints, can cause backlash [25]. The same applies for friction in the guiding system. Backlash is a position error that appears upon reversing direction and impacts both accuracy and repeatability. However, it cannot be detected from the internal measurement system. Instead, backlash can be modeled after intensive calibration [26,27,28], can be reduced by using flexure joints, prestressed, or backlash-free mechanisms [29,30], or can be measured and compensated with a direct pose measurement system.

Similar to backlash, temperature drift is a critical issue that cannot be measured with the internal measurement system. The same applies to the drift caused by mechanical stress. The main sources for temperature drift are ambient temperatures and heat caused by the motors, the drives, and the control. Depending on the materials and the length of the active joints, the temperature drift in the parallel mechanism’s pose can reach several micrometers. See, for example, [31], where a position drift of over 200 µm was obtained after eight hours for a hexapod with linear actuator lengths of 2860 mm. Temperature drift can be modeled and compensated if the heat source, the produced heat, and the materials’ thermal expansion coefficient are known. Kalas et al. [32] proposed a method to eliminate the influence of thermal deflection by measuring the temperatures of every linear actuator at a reference pose and predicting the thermal expansion of the platform.

Consider, furthermore, that the parts of the parallel mechanism are manufactured and thus vulnerable to manufacturing inaccuracies. These resulting uncertainties can only be incorporated by a huge model whose parameters need to be found by calibration. Masory et al. [33,34], for example, discovered that a full model of the general Stewart–Gough platform requires 132 parameters and extensive calibration.

In conclusion, parallel mechanisms are theoretically ideal for high-precision applications due to their high stiffness and their low moving masses; however, they can only achieve this precision if no measurement errors occur and the kinematic chains are ideal, i.e., no offsets, deformation, backlash, or misalignment appear in the kinematic links (joints, linear actuators, and platforms). This, however, is not possible in real-world applications and, consequently, precise positioning is limited by these errors.

**Figure 1:**
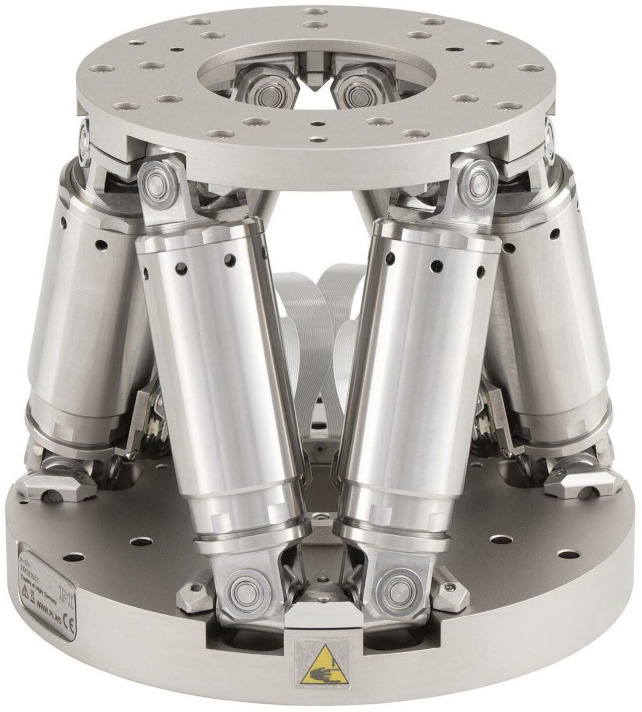
H-811.I2 6-axis miniature hexapod by Physik Instrumente (PI) GmbH & Co. KG. Picture by Physik Instrumente (PI) [35].

In order to overcome this issue, a vision-based concept for absolute pose sensing for parallel mechanisms is proposed. The idea is to include a camera on the base platform of a parallel mechanism and to attach an array of micro-structured AprilTags on the moveable manipulator platform. AprilTags are visual fiducial markers with a special shape containing a unique ID that allows a fast recognition and an absolute pose estimation. By knowing the positions of the tags on the manipulator platform, the actual pose of the moving platform can be evaluated. In this paper, such a high-precision pose measurement system is presented and applied on a state-of-the-art hexapod, the H-811.I2 6-axis miniature hexapod by Physik Instrumente (PI) GmbH & Co. KG shown in Figure 1. Furthermore, restrictions and external parameters are investigated to help design the vision-based pose measurement system for this specific application, as well as for general parallel mechanisms.

The remainder of this paper is as follows. In Section 2, the state of the art in image-based pose estimation is reviewed. In Section 3, our concept for a high-precision pose measurement system using fiducial tags is presented. This is followed by Section 4, where preliminary investigations are performed to support our concept and to help design this vision-based pose measurement system. In Section 5, the achievable accuracy of our vision-based concept for absolute pose sensing is investigated on the example of a state-of-the-art hexapod. Finally, a summary and a critical discussion is given in Section 6.

## 2. State of the Art

This section is composed of two parts. In Section 2.1, an overview about methods and concepts for image-based pose calculations is given. This is followed by Section 2.2, where applications and accuracy investigations on visual fiducial markers are reviewed.

### 2.1. Direct Image-Based Pose Calculation

As mentioned above, direct pose measurements offer several advantages since the pose of the moveable manipulator platform is directly measured and all the changes in the pose (for example, due to deformation or backlash in the joints) can be directly observed and later controlled. An excellent example for the need of high-precision direct pose measurements are magnetically levitated positioning stages that shall be extremely accurate to position wafers for the semiconductor industry [36]. Here, the moveable stage is directly measured (see, for example, [37]), but a high number of incremental and analog sensors is required.

Another possibility for a direct image-based pose calculation is photogrammetry, where a 3D object is reconstructed from a set of images taken from different points of views; see, for example, [38,39]. Disadvantages of this concept for high-precision pose measurements are the huge amount of data that is required for obtaining a complete 3D model of the scanned object and the complex and time-consuming computation. Furthermore, for a highly accurate pose measurement, cameras with an extremely high resolution are required.

The basis for direct image-based pose calculations is solving the perspective-n-point (PnP) problem, where the pose of an object is computed with respect to the camera from a set of image points [40,41]. A systematic investigation of this problem can be found in [42]. It was shown that the P3P problem (with three pairs of object and image points) can have up to four solutions that can then be disambiguated by using a fourth point. In recent years, new concepts for solving the P3P problem were proposed that improve computing time and numerical stability; see, for example, [43,44,45].

For parallel mechanisms, direct pose measurements offer huge opportunities, as mentioned above. However, using a camera to observe the moveable manipulator platform is comparably rare. In fact, Baron and Angeles [46] were the first to propose using a camera that is mounted on the fixed base platform in addition to the six length sensors to solve the direct kinematics problem of the Stewart–Gough platform. In 2019, Moldagalieva et al. [47] used the improvements in visual fiducial tags in terms of recognition and pose detection to estimate the pose of a tensegrity manipulator. Using tag sizes of 5.6 cm and a distance between the camera and the tags of 1.5 m, Moldagalieva et al. [47] realized a RMS position error of 2.3 cm with a standard deviation of 1.4 cm and a RMS orientation error of 7.5${}^{\circ }$ with a standard deviation of 3.14${}^{\circ }$. Kuzdeuov et al. [48] further improved this concept by adding a feed-forward neural network and a sensor fusion concept. However, accuracies of less than 1 cm were not achieved. Zake et al. [49] evaluated concepts for the pose estimation of a cable-driven parallel robot. As one of their investigated concepts, they installed a camera on the moving platform of the robot and used a fixed AprilTag. However, using their vision-based concept, they obtained the worst results in their experiments, with position errors of up to 5 cm and orientation errors of up to 12${}^{\circ }$.

In the field of robot localization (on the ground [50,51,52], on and under water [53,54,55], as well as in the air [56,57,58]) and calibration [59,60,61], pose calculation from visual fiducial tags plays an important role. For localization purposes, however, the robots usually have working ranges of several centimeters or even meters, so that smaller errors in the micro- or nanometer range are not significant in these applications or do not significantly affect the results of the pose detection. In fact, the sizes of the visual fiducial tags range from 5.6 cm (in [47]) to 0.7 m (in [54]) in the previously mentioned applications, while the distance between camera and object range from 0.4 m (in [58]) to 30 m (in [54]).

### 2.2. Visual Fiducial Markers

**Figure 2:**
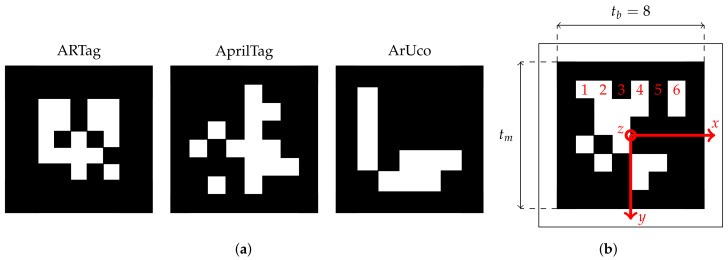
Types and structures of different visual Fiducial tags: (**a**) ARTag (left), AprilTag (center), and ArUco (right), and (**b**) shape, general structure, and coordinate system of an AprilTag of family 36h11 (here: ID 0).

For image-based pose calculations, visual fiducial markers are essential as a reference system whose lengths, shape, and structure is known. Nowadays, fiducial tags such as ARTags, AprilTags, or ArUcos are used (see Figure 2a), that all have their strengths and weaknesses as investigated, for example, in [62,63,64,65,66]. In this work, AprilTags are used as they perform comparably well in terms of accurate and robust pose detections; see, for example, [67].

AprilTags [68,69,70] are special types of visual fiducial tags such as barcodes or QR-codes. Unlike these, which are designed for a wide range of user-defined information such as web links, product information, or prices, AprilTags are specifically designed for fast and reliable recognition and identification even at large distances. In fact, AprilTags have a special shape and appearance to make them distinguishable from each other and to contain as little data as possible so that their precise position and orientation with respect to the camera can be decoded as quickly as possible—an immanent requirement for real-time robotics application. AprilTags are squared visual fiducial tags with a black and white frame whose corners are used for pose estimation. The frame’s width corresponds to one bit. In the center of the tag, the bits are arranged according to a specific pattern, depending on the tag family. A tag family, consisting of several unique and distinguishable tags, is characterized by two numbers: the number of data bits in the center of the tag and the hamming distance between adjacent tags, i.e., the distance to avoid faulty identifications. For example, the family 36h11 is a 6-by-6 data square with a hamming distance of 11 resulting in 587 different unique tags. Figure 2b shows an AprilTag of this family.

The localization accuracy that can be achieved with AprilTags depends on several factors. Abbas et al. [63], for example, analyzed the position error propagation and identified the angular rotation of the camera about its vertical axis as the primary source of error. Kallwies et al. [64] extensively studied the localization accuracy of AprilTags and concluded that AprilTags with a size of 10×10 px (i.e., camera pixels) can be detected with certainty. The mean error was 0.25 px and has a maximum of 1.17 px. By using edge refinement, the error was reduced to a mean error of 0.1 px with a maximum of 1.17 px. Cesar et al. [53] compared AprilTags with other available marker systems such as ARToolKit and ArUco in an underwater terrain and for two lighting scenarios. They showed that even for underwater application, AprilTags with a size of 21×21 px can be correctly detected.

The corners of AprilTags are similarly detected as the corners of a checkerboard pattern that is usually used for camera calibration, namely, by the intersection point of two intersecting lines. According to Hartley and Zisserman [71], therewith, a tag detection accuracy of 0.1 px can be obtained. Detection errors of corner points in the image plane generate errors in the object plane:

(1) ω·d=f·Δ,

where *d* is the detection error of the corner point in the image plane, Δ the resulting error of the corner point in the object plane, *f* is the focal length of the camera, and ω is the distance of the object to the camera.

## 3. Concept for a Vision-Based Pose Detection

**Figure 3:**
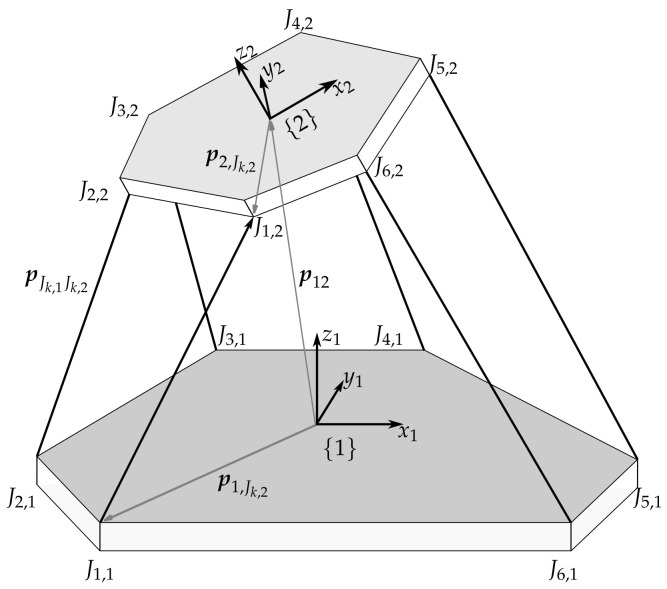
General assembly and nomenclature of the Stewart–Gough platform.

Consider a parallel mechanism such as the Stewart–Gough platform (shown in Figure 3) consisting of a fixed base platform, a moveable manipulator platform, base and manipulator platform joints, as well as six linear actuators. Each platform has its own body-fixed coordinate system including the base platform {1} and the manipulator platform {2}. The vector from the center of the platform to the fulcrum of the *k*th joint $J_{k,i}$ of platform {i} is denoted as $p_{iJ_{k,i}}$ and the connection vector between the joints $J_{k,1}$ and $J_{k,2}$ of platforms {1} and {2} as $p_{J_{k,1}J_{k,2}}$ with k∈{1,…,6}. Using inverse kinematics, this vector is given by

(2) $$\begin{array}{c} {}^{1}p_{J_{k,1}J_{k,2}}={}^{1}p_{12}+{}^{1}R_{2}\cdot {}^{2}p_{2,J_{k,2}}-{}^{1}p_{1,J_{k,2}} \end{array}$$

with respect to platform {1}. Here, ${}^{1}R_{2}$ denotes the rotation matrix from frame {2} into frame {1}, and ${}^{1}p_{12}$ is the vector connecting the origins of platforms {1} and {2}. Therewith, the pose is given by the rotation matrix ${}^{1}R_{2}$ and the position vector ${}^{1}p_{12}$. The parallel mechanisms’ pose, however, cannot be calculated unambiguously, and usually not analytically, from the linear actuators’ lengths. In order to solve this problem as well as to overcome the above-mentioned limitations and instead be able to measure, identify, and compensated all the undesired motions without extensive calibrations, in the following, our vision-based concept for absolute pose sensing for parallel mechanisms is presented.

### Vision-Based Concept for Pose Detection

**Figure 4:**
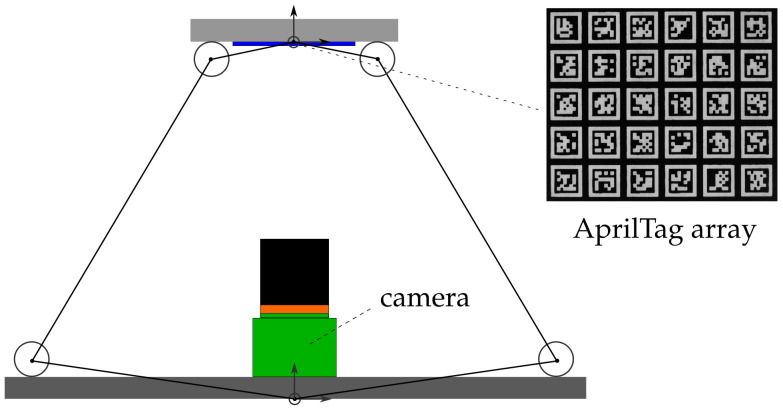
Vision-based concept for pose detection: Side view of a parallel mechanism with a camera on the base platform and an AprilTag array on the bottom side of the manipulator platform.

Consider a parallel mechanism where an array of AprilTags is mounted on the bottom side of the moveable manipulator platform. Consider furthermore a camera that is mounted in such a way on the base platform that the camera axis coincides with the *z*-axis of the base platform. In order to obtain a high position accuracy, the camera’s field of view (FOV) is very small, so that only a section of the AprilTag array is visible. The AprilTags’ sizes are chosen accordingly and they are arranged uniformly on the array in such a manner that, for every pose of the parallel mechanism, at least one AprilTag is completely visible by the camera. Knowing the location of each AprilTag on the array, for example by storing them in an look-up table, and furthermore being able to detect, identify, and compute its pose for any AprilTag that is seen by the camera, it is possible to obtain the parallel mechanism’s pose for every possible pose in the workspace. Figure 4 illustrates this concept.

In order to obtain a high-precision pose measurement system from this vision-based concept, there are several issues that need to be considered in advance. At first, the parallel mechanism’s workspace and the camera’s depth of field are counterparts where a trade-off needs to be found. In fact, the camera’s depth of field needs to be at least as big as the parallel mechanism’s workspace in *z*-direction. As the camera’s depth of field is directly related to the chosen objective lens and the distance between the camera and the observed object, a minimum distance for the camera with respect to the manipulator platform can be found. A second issue is the AprilTag array. The array must have a minimum size so that it is visible by the camera at any pose of the manipulator platform. Furthermore, the AprilTags themselves require a minimum size so that they can be detected and identified by the camera. Finally, the maximum distance between the AprilTags must not exceed a limit so that at least one AprilTag remains in the camera’s field of view.

## 4. Design of a High-Precision Pose Measurement System for Parallel Mechanisms

As mentioned in the previous section, several parameters influence and restrict the achievable accuracy of our vision-based sensor concept including the parallel mechanism, the chosen camera system, the lightning, and the PnP algorithm. In order to obtain a high-precision pose measurement system, in the following, the design of such a measurement system for parallel mechanisms is explained step by step. Furthermore, the design phases are illustrated on the H-811.I2 6-axis miniature hexapod as an ongoing application example. The experiments performed in this section are explained in more detail in Appendix A.

### 4.1. Boundary Conditions

As mentioned above, the parallel mechanism and its workspace represent physical restrictions for the pose measurement system. The mechanism’s workspace in *x*- and *y*-direction determines the required size of the AprilTag array to ensure that at least one AprilTag is always visible in the camera’s field of view. On the other hand, the mechanism’s workspace in *z*-direction determines the required depth of field. In addition, the camera’s working distance must be smaller than the distance between the manipulator platform and the base platform of the parallel mechanism in its home position, so that the camera system can be integrated.

#### Application Example: Workspace and Boundary Conditions of the H-811 Hexapod

The hexapod, shown in Figure 1, has a translational travel range *T* of ±17 mm, ±16 mm, and ±6.5 mm in *x*-, *y*-, and *z*-direction, respectively, a rotational travel range of ±10${}^{\circ }$ around the *x*- and *y*-axis, and a rotational travel range of ±21${}^{\circ }$ around the *z*-axis. In its home position, the hexapod has a height of 114.3 mm from the top of the manipulator platform to the bottom of the base platform. The manipulator and base platform have a height of 17 mm and 9 mm, respectively. The hexapod has an aperture hole with a diameter of 50 mm in the manipulator platform and 40 mm in the base platform. The outer diameter of the base and manipulator platform are 100 mm and 136 mm, respectively.

**Figure 5:**
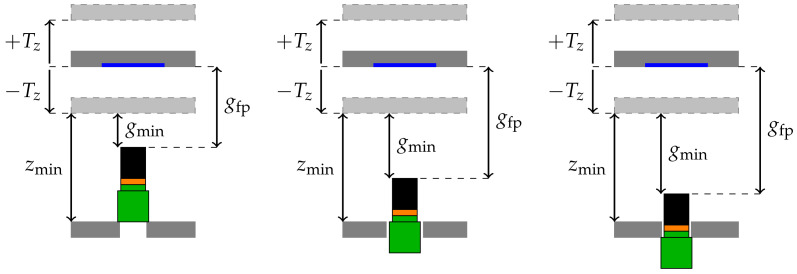
Possible integrations of the camera system in the hexapod.

Knowing these limits, the camera can be installed in the following ways, see Figure 5:

1. The entire camera system is located inside the hexapod.
2. The camera is located within the aperture hole of the base platform. The objective lens is either located partially within and partially above the aperture hole of the base platform or it is entirely above the base platform.
3. The camera is located completely below the base platform and the objective lens is inside the aperture hole and partially above the base platform.
4. The entire camera system is located below the base platform of the hexapod.

Possibility 4 (not shown) is not suggested as the entire system height becomes very high. The final choice of the camera system integration mainly depends on the camera’s minimal working distance and its depth of field. In fact, the hexapod’s travel range in *z*-direction $T_{z}$ should coincide with the camera’s depth of field. Similarly, the distance between the camera and the manipulator platform *g* at the hexapod’s home position should coincide with the camera’s focal plane $g_{\mathrm{fp}}$. The minimum distances between base and manipulator platform and the camera’s minimal working distance are denoted as $z_{\min}$ and $g_{\min}$.

### 4.2. Camera System

The camera system is selected based on the boundary conditions given by the parallel mechanism. It consists of an industrial camera, an objective lens, and possibly a spacer. The camera sensor has a pixel size px and a resolution $RES_{x}$ in *x*-direction and $RES_{y}$ in *y*-direction. The sensor dimensions $L_{x}^{sensor}$ and $L_{y}^{sensor}$ are calculated with

(3) $$\begin{array}{ccc} L_{x}^{sensor}=RES_{x}\cdot px\;, & \; & L_{y}^{sensor}=RES_{y}\cdot px\;. \end{array}$$

The objective lens has a focal length *f* and a minimal object distance of $g_{\mathrm{fp}}$. For a given working distance *g*, the resulting field of view $FOV_{x}$ and $FOV_{y}$ in *x*- and *y*-direction can be calculated as follows:

(4) $$\begin{array}{ccc} FOV_{x}=\left(\frac{g}{f}-1\right)\cdot L_{x}^{sensor}\;, & \; & FOV_{y}=\left(\frac{g}{f}-1\right)\cdot L_{y}^{sensor}\;. \end{array}$$

If a spacer is used, the new working distance $g^{\prime }$ can be calculated from the lens equation:

(5) $$\begin{array}{cc} g^{\prime }={\left(\frac{1}{f}-\frac{1}{b+w}\right)}^{-1}\;, & \;\mathrm{with}\;b={\left(\frac{1}{f}-\frac{1}{g_{\mathrm{mp}}}\right)}^{-1}\;, \end{array}$$

where *w* is the spacer width and *b* is the image distance which is obtained from camera calibration; see Section 5.2. Note that the manufacturer usually specifies the working distance $g_{\mathrm{fp}}$ with respect to the front of the objective lens and not the optical main plane. The connection between them is given by $g_{\mathrm{mp}}=g_{\mathrm{fp}}+\left(l-b\right)$, where $g_{\mathrm{mp}}$ is the working distance with respect to the optical main plane, $g_{\mathrm{fp}}$ the distance to the objective lens, and *l* is the distance between camera sensor and objective lens.

From this, the smallest detectable unit ε can be calculated:

(6) $$\begin{array}{c} \varepsilon =\eta \cdot spx\cdot \frac{FOV_{x}}{RES_{x}}=\eta \cdot spx\cdot \frac{FOV_{y}}{RES_{y}}\;, \end{array}$$

where η is the Nyquist factor and spx is a parameter indicating subpixeling. For monochrome cameras, η=2 can be selected. For color cameras, an RGB pixel consists of several individual pixels with a corresponding color filter. Consequently, η≥3 must be used. If no subpixeling is assumed, spx=1 can be selected, whereas subpixeling factors of 0.1 can be achieved with modern software methods [72].

**Figure 6:**
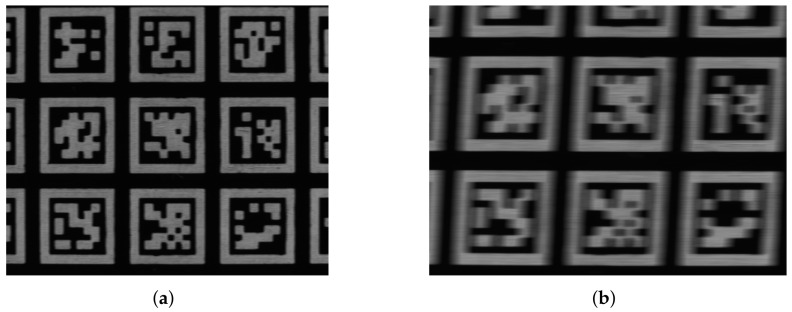
Camera image of moving objects captured (**a**) with a global and (**b**) a rolling shutter.

For dynamic measurements, the camera’s shutter mechanism is crucial. Using a global shutter, the exposure of each pixel ends simultaneously so that moving objects can be captured without distortion. With a rolling shutter, on the other hand, different lines of the sensor are exposed at different times, leading to distortion when capturing moving objects. Figure 6 shows camera images of moving objects captured with a global shutter and with a rolling shutter. It can be observed that the picture captured with the rolling shutter has a definite distortion and motion blur. This is not the case for the global shutter. Consequently, a camera with global shutter should be used for dynamic pose detections, whereas, for static observations, a camera with a less expensive rolling shutter is also sufficient.

#### Application Example: Camera System for the H-811 Hexapod

For the hexapod, a monochrome camera with 5 MP resolution (2448 × 2048) and a pixel size of 3.45 µm (MER2-503-36U3M by Daheng Imaging) as well as a ultra high-resolution objective lens with a focal length of 35 mm is used (LCM-10MP-35MM-F2.8-1.5-ND1). The camera has a global shutter. By using a 10 mm spacer, the objective lens’ minimal working distance can be reduced from 100 mm to 48 mm. Given the pixel size px of 3.45 µm, a resolution of 2448 × 2048, and a focal length f=35 mm at a working distance of g=48 mm (reduced by spacer), the smallest unit ε that can be detected by the camera system can be calculated:

(7) $$\begin{array}{c} \varepsilon =2\times 1\times \frac{13.04\;\mathrm{mm}}{2448}=10.65\;µm\;. \end{array}$$

Considering a reasonable subpixeling factor of 0.1, units of 1.065 µm can be detected for the given working distance.

### 4.3. Design of the AprilTag-Array

**Figure 7:**
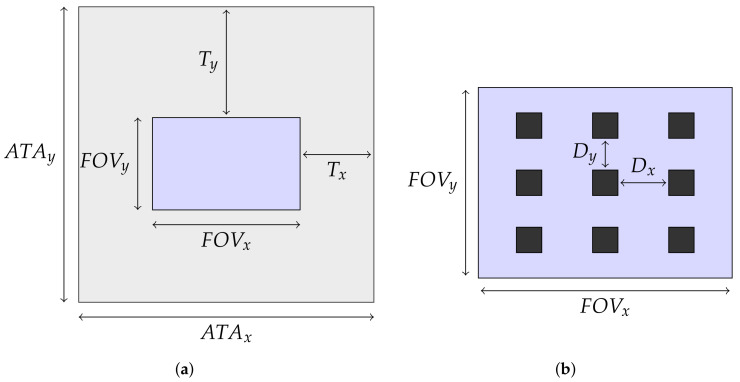
Graphical representation of the camera’s field of view (blue), the AprilTag array (gray), AprilTags (black), and their arrangement in the the camera’s field of view: (**a**) Parameters determining array dimensions, and (**b**) AprilTag arrangement.

As mentioned above, the size of the AprilTag array ($ATA_{x}$ and $ATA_{y}$) is given by the camera’s field of view and the parallel mechanism’s workspace. It can be calculated as follows:

(8) $$\begin{array}{ccc} ATA_{x}=FOV_{x}+2\cdot T_{x}\;, & \; & ATA_{y}=FOV_{y}+2\cdot T_{y}\;, \end{array}$$

where $T_{x}$ and $T_{y}$ are the parallel mechanism’s travel ranges in *x*- and *y*-direction. Within this array, there needs to be a number of AprilTags whose size and location, i.e., the distance between them ($D_{x}$ and $D_{y}$), depends on the camera choice. Figure 7 shows the relation between the camera’s field of view, the AprilTag array, and the required distances between the AprilTags. The size of the AprilTags must lie between two boundaries. The lower limit is given by the minimum size of an AprilTag that can only just be detected from the camera. The upper limit is given by the maximum size of an AprilTag, that should be smaller than the camera’s field of view. If more than one AprilTag shall be seen in the camera’s field of view, its maximum size reduces accordingly. In the following, the procedure to calculate the minimum size of an AprilTag is presented.

For the minimum size, the AprilTags have to be detected reliable at any given distance *g* in the workspace of the parallel mechanism. The minimum tag size $t_{\min}$ where tags are still detected can be calculated according to the Nyquist–Shannon sampling theorem with

(9) $$\begin{array}{c} t_{\min}=px\cdot p\cdot t_{b}\cdot \frac{g_{\max}-f}{f}\;, \end{array}$$

where $t_{\min}$ is the minimum tag size $t_{m}$ in meters, px is the camera’s pixel size, *p* is a sampling factor that corresponds to the number of pixels that are needed to detect one bit ($p_{\min}=2$), $t_{b}$ is the size of the tag in bits (number of modules), and the fraction represents the inverse mapping scale. In this context, $g_{\max}$ is the maximum distance between camera and AprilTag array and corresponds to the parallel mechanism’s maximum travel range in *z*-direction (i.e., $g_{\max}=g_{\mathrm{fp}}+T_{z}$).

As the parallel mechanism moves, the AprilTag array moves in the camera’s field of view. Consequently, it is not sufficient to have only one AprilTag on the array as this cannot be seen for every pose of the parallel mechanism. In order to guarantee that at least one AprilTag is always in the camera’s field of view, multiple AprilTags are needed that must have a specific distance between them. The horizontal and vertical distance between two adjacent AprilTags can be calculated as follows:

(10) $$\begin{array}{ccc} D_{x}=\frac{FOV_{x}-\left(n_{x}+1\right)\cdot t_{m}}{n_{x}}\;, & \; & D_{y}=\frac{FOV_{y}-\left(n_{y}+1\right)\cdot t_{m}}{n_{y}}\;, \end{array}$$

where $t_{m}$ is the tag size in meters. The number of AprilTags that are seen in the camera’s field of view is specified by $n_{x}\times n_{y}$.

#### Application Example: AprilTag-Array Design for the H-811 Hexapod

Knowing that the camera’s field of view is 13.03 mm × 10.91 mm and the hexapod’s travel range is ±17 mm, in *x*-direction and ±16 mm in *y*-direction, the required AprilTag array size is 47.03 mm in *x*-direction and 42.91 mm in *y*-direction. Due to the smaller diameter of the aperture hole in the manipulator platform (Ø 50 mm), for the hexapod, a squared AprilTag array with an edge length of 35 mm is used. On the array, nine times nine AprilTags of the family 36h11 and an edge length of 2.4 mm are arranged on the bottom side of the hexapod’s manipulator platform. The AprilTags are uniformly distributed on the array with a horizontal and vertical distance of 1.3 mm. Consequently, at least two times two AprilTags are seen in the camera’s field of view. The AprilTag array is made of alloy and the AprilTags are laser-engraved on a black laser foil.

**Figure 8:**
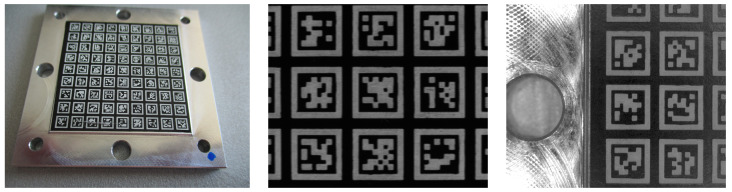
Manufactured AprilTag array: (**left**) photography of the AprilTag array, (**center**) general image of the camera’s field of view, and (**right**) image of the camera’s field of view at the edge of the working area.

Figure 8 shows the manufactured AprilTag array as well as an image of the camera’s field of view where at least two times two, but not more than three times three, AprilTags can be seen. Using a shorter AprilTag array size, fewer tags are visible at maximum displacement because the field of view is not completely covering the array. Nevertheless, at least one AprilTag can still be seen; see Figure 8.

### 4.4. Influence of the Working Distance

The camera system has a given minimal working distance and objects below this distance, in general, cannot be detected sharply. However, the working distance must be small enough, so that the camera system can be implemented inside of the parallel mechanism or as close to the manipulator platform as possible to prevent any occlusion. Furthermore, a shorter working distance leads to a smaller field of view and therefore a higher magnification of the AprilTags. Consequently, this would increase the resolution and the achievable accuracy. For this reason, the working distance shall be reduced, which is generally achieved by using spacers. Spacers, however, can lead to increased distortion, higher chromatic and spherical aberration, and unequal illumination. Furthermore, a smaller working distance results in a smaller depth of field that reduces the effective working range. In consequence, a compromise must be found between accuracy and working range.

In the following, the influence of spacers on the camera’s depth of field is analyzed. The camera’s depth of field is mainly influenced by the working distance, i.e., the distance between the camera and the observed object. The shorter the working distance, the smaller the camera’s depth of field. The camera’s depth of field can be increased again by a wider shut aperture but this would require a longer exposure time.

**Figure 9:**
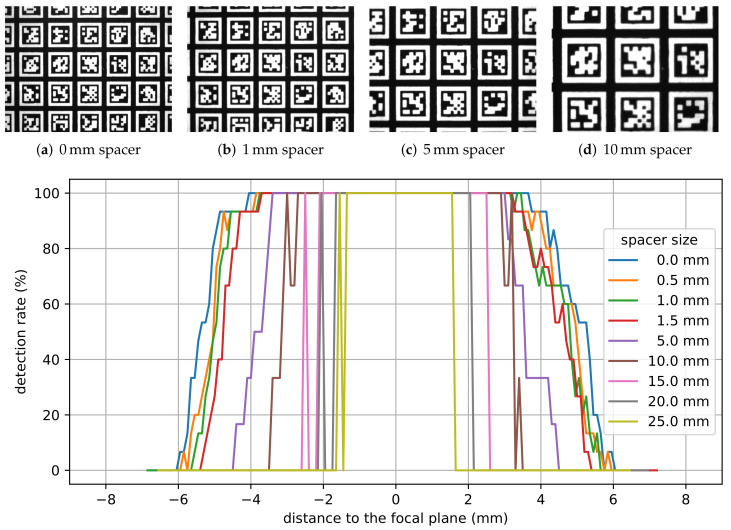
Spacer size and the corresponding fields of view in the focal plane: (**a**) 0 mm, (**b**) 1 mm, (**c**) 5 mm, and (**d**) 10 mm spacer. Influence of the spacer size on the detection rate of AprilTags.

Figure 9a–d show the corresponding fields of view in the focal plane for different spacer sizes when looking at the AprilTag array shown in Figure 8. One can clearly see the magnification of tags. In fact, 15 to 20 AprilTags can be seen without a spacer, whereas only three to six AprilTags are entirely visible in the camera’s field of view when using a 10 mm spacer. Figure 9 furthermore shows the influence of the spacer size on the detection rate of AprilTags for different distances to the camera’s focal plane. A detection rate of 100% means that every AprilTag that is visible in the camera’s field of view is detected, whereas lower detection rates indicate that one or more AprilTags were not detected properly. Due to the camera’s depth of field, for increasing distances to the focal plane, the detection rate should decrease. It can be noticed that, for wider spacers, the detection rate deteriorates for lower distances than for smaller spacers. In fact, while the detection rate is still 100% for the camera system without a spacer for distances ±4 mm to the focal plane, for the camera system with the 10 mm spacer, the detection rate is already at 0% at this distance. With this spacer, detection rate of 100% is only possible within ±3 mm to the focal plane.

**Figure 10:**
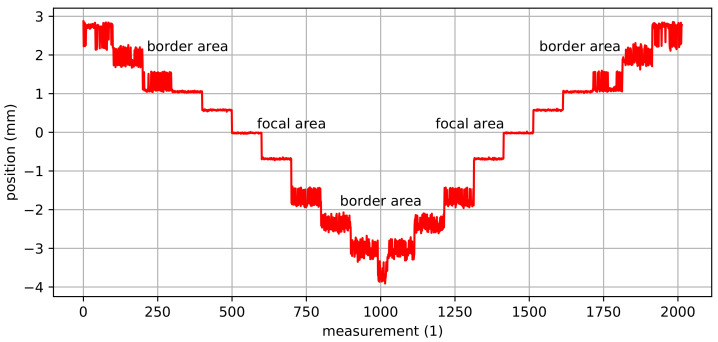
Estimated poses for a staircase motion in *z*-direction with a step size of 0.6 mm where AprilTags are detected in the border area as well as in the focal area.

Due to the camera’s depth of field, AprilTags in the focal area are detected more accurately than AprilTags in the border area. The border area is the area with a distance to the focal plane where the detection rate starts to deteriorate but AprilTags are still detected. Consequently, the parallel mechanism’s pose is estimated to be less accurate in the border area than in the focal area. Figure 10 shows the estimated poses for a staircase motion in *z*-direction where AprilTags are detected in the border area as well as in the focal area. It can be noticed that the poses in the border area are significantly more noisy than in the focal area. Furthermore, not all AprilTags were detected (indicated by missing measurements).

#### Application Example: Spacer for the H-811 Hexapod

For the Hexapod, a 10 mm spacer is used. This reduces the camera’s working distance from 100 mm down to 48 mm. Furthermore, an additional magnification of the AprilTags is achieved as the tags are displayed larger in the camera’s field of view and consequently larger on the camera’s sensor. In fact, the magnification of the AprilTags leads to a higher sampling of the corner points so that the hexapod’s pose can be estimated more accurately and robustly. The spacer, on the other hand, reduces the camera’s depth of field down to ±3.5 mm around the focal plane.

**Figure 11:**
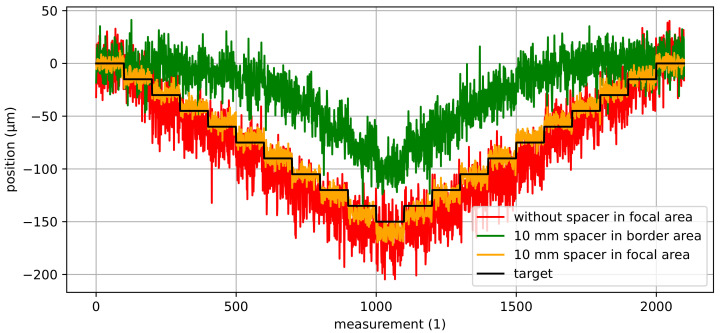
Comparison of the estimated poses with a 10 mm spacer and without for a staircase motion in *z*-direction with a step size of 15 µm in the focal and border area.

Figure 11 shows a comparison of the estimated poses without and with a 10 mm spacer for a staircase motion in the focal area in *z*-direction with a step size of 15 µm. It can be noticed that the pose estimates from the camera system with the 10 mm spacer are significantly more accurate compared to the ones without a spacer. In fact, with a 10 mm spacer, the measurement noise is three times lower than the noise without a spacer. Consequently, the mean values are much closer to the target values for the camera system with the 10 mm spacer. As the camera’s depth of field also influences the results, the estimated poses with a 10 mm spacer in the border area are additionally shown in Figure 11. The estimated poses with a 10 mm spacer in the border area have similar noise compared to the poses obtained without a spacer in the focal area. However, the position accuracy is significantly worse. In fact, neither the staircase motion nor actual steps can be identified with certainty.

### 4.5. Illumination

**Figure 12:**
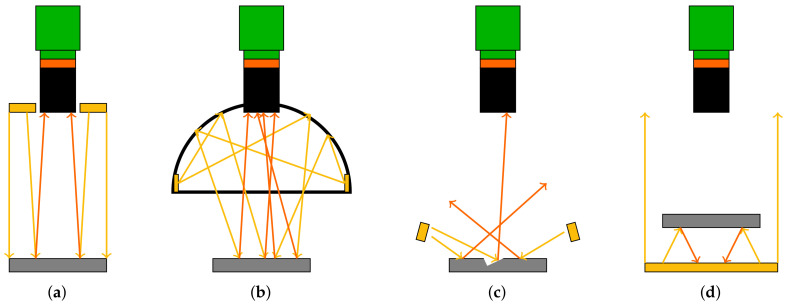
Different lighting techniques: (**a**) direct incident lighting, (**b**) diffuse lighting, (**c**) dark field illumination, and (**d**) transmitted light illumination.

In order to obtain optimal image data, homogeneous lighting conditions must be maintained continuously. Illumination can be controlled externally by choosing an appropriate lighting technique or with the camera system itself via the aperture or the exposure time. In the first case, the angle of the incident light plays a decisive role in the illumination and influences which features of the object are reflected and thus visible. Based on the position of the lighting relative to the camera and the angle of illumination, a distinction can be made between different lighting techniques. These are shown schematically in Figure 12.

With direct incident light, the illumination is arranged vertically to the camera. For this purpose, ring lights can be used. This technique is well suited for non-reflective materials. If reflective surfaces such as metallic objects or polished surfaces are observed, it is necessary to use diffuse light. Dome or coaxial illumination can be used in this case. With dark field illumination, the observed object is illuminated at a low angle. This causes the light to be reflected away from the camera so that the object remains dark. In case of irregularities on the object, they reflect the light in the direction of the camera and they become visible. Dark field illumination is therefore well suited for the detection of slanted edges, scratches, imprints, slits, and elevations. In contrast to incident light illumination, transmitted light illumination lightens the observed object from the opposite side of the camera, i.e., below the object. Due to the high illumination intensity, this technique is resistant to scattered light. It is particularly well suited for accurate size measurements of parts and objects.

In addition to the external lightning, it is also possible to control the illumination of the image via the aperture or the exposure time of the camera system. The aperture can be used to vary the opening of the objective lens and thus the incident light. The depth of field increases the further the aperture is closed, but simultaneously a longer exposure time is required. The exposure time is the time where the shutter is opened and the camera sensor is illuminated. The shorter the exposure time, the higher sampling rates of the camera can be achieved. Shorter exposure times are thus favorable but insufficient external lightning would lead to a reduced contrast in the image and can cause faulty detections.

#### Application Example: Illumination for the H-811 Hexapod

For the hexapod, a ring light with 1.4 W power, a 40 mm diameter, and twelve circular arranged LEDs is mounted on top of the objective lens. In order to measure under homogenous conditions, the hexapod is completely covered. For this application, direct incident lighting is not ideal as mentioned before, but transmitted light illumination is not possible because the manipulator platform needs to be passive, i.e., no heat source should be placed on it. For pose measurements, an aperture stop of f/8 and an exposure time of 3 ms is used. The aperture blades reduce the aperture diameter of the optics by a factor of $\sqrt{2}$, which corresponds to halving the aperture area. An aperture stop of f/8 indicates that one eighth of the aperture area is open.

Especially in the context of AprilTags, illumination is crucial and does not only affect the detection of AprilTags but also the achievable accuracy of pose estimates. Reconsider that AprilTags have squared outer frames whose corners are used for pose estimation. In case of direct incident lighting, a higher illumination increases the contrast between the black and white bits but it also leads to an overabundance of light that causes the white areas of the image to bloom out into the surrounding areas. This, however, causes variations in the detected corner points which in turn result in inaccurate pose estimates. The phenomenon is presented in [54].

**Figure 13:**
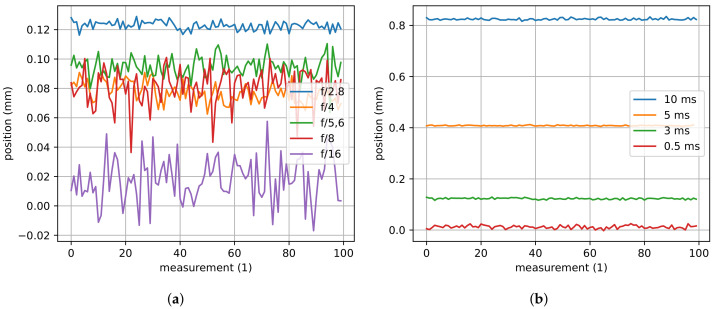
Influence of the illumination on the pose estimate in *z*-direction: (**a**) constant exposure time of 3 ms with variable aperture stops and (**b**) constant aperture stop f/8 with variable exposure times.

Figure 13 shows the influence of illumination on the estimated pose of the hexapod. In this experiment, the hexapod is held at a static position (here, its home position), the exposure time is set to 3 ms, and the aperture stop is set to f/8. Thereafter, the exposure time and the aperture stop are varied one at a time while the other one is held constant. It can be noticed that the aperture stop does affect the pose estimate in *z*-direction but the effect is comparably small. In fact, varying the exposure time significantly changes the pose estimate. Nevertheless, the aperture stop changes the measurement noise. While an f/2.8 aperture stop has a measurement standard deviation of 2.86 µm, an aperture stop of f/16 has a standard deviation of 15.03 µm. In contrast to the aperture stop, the exposure time does not seem to affect the measurement noise. Here, the mean pose is shifted due to the above-mentioned blooming effect. While an exposure time of 0.5 ms leads to a mean pose estimate of −0.07 mm, an exposure time of 10 ms leads to a pose shift of 0.81 mm (pose estimate of 0.74 mm).

### 4.6. PnP Algorithms

In order to compute the parallel mechanism’s pose from the four corner points of an AprilTag, the PnP problem needs to be solved. As mentioned before, there are several formulations to solve this problem. As input, the known object points (stored, for example, in a look-up table) and the detected corner points of the AprilTags are required. From this, they solve the PnP problem to obtain the pose of the observed AprilTags.

The AprilTag algorithm is already implemented in the AprilTag library and was described in [68,69]. The AprilTag implementation uses a DLT-algorithm, a singular value- and a QR-decomposition with subsequent orthogonal iteration to improve the estimation. As an alternative, the IPPE (infinitesimal-plane-based pose estimation) algorithm [73] can be used, which is based on the idea of determining the pose by a transformation over an infinitesimally small region on the surface. It exploits the fact that in noisy homographs, the true transformation between the model plane and the image is better in some regions of the plane than in others. The method is thus based on finding the point where the transformation is best estimated and using only this local information to constrain the pose. The SQPnP (sequential quadratic PnP) algorithm [74] formulates the PnP problem as a nonlinear quadratic program and identifies regions in the parameter space that contain unique minima. This guarantees that at least one of them is the global minimum. Each local minimum is computed using a sequential quadratic programming (SQP) method. The ITERATIVE algorithm is based on a Levenberg–Marquardt optimization. Here, the algorithm finds a pose that minimizes the reprojection error. This error is defined as the sum of the squared errors between the image points and the object points projected onto the image plane. The AP3P (algebraic P3P) algorithm [75] provides an algebraic solution to the classical P3P problem. For this purpose, a system of trigonometric equations is set up and solved via an algebraic approach. EPNP [76] is a non-iterative algorithm where the 3D points are represented as a weighted sum of four virtual control points. The problem thus reduces to estimating these coordinates in the camera’s coordinate system.

#### Application Example: PnP Algorithm for the H-811 Hexapod

For the hexapod, the implemented AprilTag algorithm is used. In order to evaluate its quality in terms of pose estimation, it is compared with the above-presented algorithms for solving the PnP problem. Therefore, a staircase motion in *x*-direction with a step size of 0.5 µm is performed, from which the detected corner points are accumulated and fed to the various PnP algorithms.

**Figure 14:**
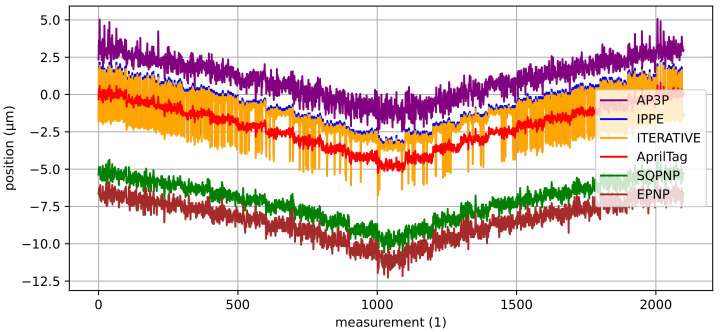
Comparison of various PnP algorithms on the achievable translational accuracy during a staircase motion in *x*-direction with a step size of 0.5 µm.

Figure 14 shows the results of this experiment. It can be noticed that the AprilTag implementation provides the best results with the minimum deviation from the target steps and the minimum measurement noise. The SQPnP and the EPnP algorithm are the best alternatives as they have similarly small measurement noise. However, they show a position deviation of −5.31 µm and −6.71 µm compared to the AprilTag algorithm. Other investigated PnP algorithms show significantly higher measurement noise (especially the IPPE and the ITERATIVE algorithm).

### 4.7. Data Handling

Before the presented vision-based sensor concept can be used as a measuring device within a control loop or other systems, it is necessary to determine the achievable sampling rate or execution time. For this purpose, the influence of the AprilTag algorithm’s parameters and the number of AprilTags in the camera’s field of view on the sampling rate is investigated.

Within the AprilTag algorithm, the parameters QD (quad decimate) and NT (number of threads) can be set. They have a significant influence on the sampling rate. QD, in this case, represents a decimation of the image resolution. For example, a QD value of two halves the resolution of the image. NT, on the other hand, corresponds to the number of threads provided to the algorithm and is an allocation of computing power. In addition to the execution time of the PnP algorithm, the duration of the image acquisition by the camera must also be taken into account. This includes the exposure time and the time for capturing and sending the image data to the computer. Last, but not least, the number of AprilTags in the camera’s field of view has a huge impact on the achievable sampling rate. The more AprilTags are detected, the more often the algorithm needs to solve the PnP problem and estimates the AprilTag’s pose.

#### Application Example: Data Handling for the H-811 Hexapod

For the Hexapod, QD is set to two and NT is set to ten. The test to evaluate the influence of the parameters of the sampling rate of the AprilTag algorithm is carried out on an Intel Core i7-10850H 2.7GHz PC with six cores. In the experiments, the parameters QD, NT, and the number of AprilTags in the camera’s field of view are set to QD = 1 (full resolution), NT = 1, and nine AprilTags. Each parameter is then systematically varied while the others are taken constant.

**Figure 15:**
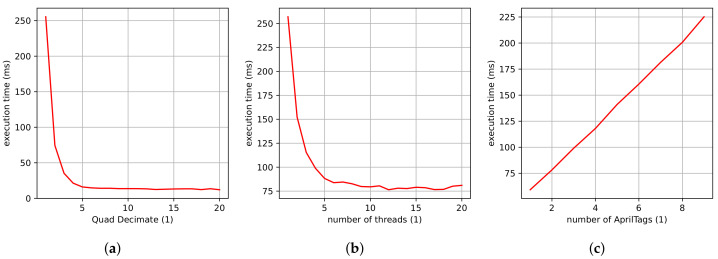
Impact on the algorithm’s execution time: (**a**) different quad decimate values where one thread is used and nine AprilTags are in the camera’s field of view, (**b**) different numbers of threads where quad decimate is set to one and nine AprilTags are in the camera’s field of view and (**c**) different numbers of AprilTags in the camera’s field of view where quad decimate is set to one and one thread is used.

Figure 15 shows the obtained results. It can be noticed that the number of AprilTags in the camera’s field of view has a linear impact on the execution time of the algorithm. This is reasonable as, for every AprilTag, the PnP algorithm has to be solved once more. The parameters QD and NT have an inverse relationship on the execution time of the algorithm. This means that the higher the parameters QD and NT are chosen, the faster the algorithm executes and the higher the sampling rates possible.However, a higher QD value leads to a lower accuracy in the position detection as well as a slight decrease in the detection rate. The exposure time and the time for capturing and sending the image data to the computer are constant, with 3 ms and 27 ms, respectively. Consequently, a sampling rate between 3.5 and 30 measurements per second can be achieved.

### 4.8. Data Fusion

As multiple AprilTags can be found in the camera’s field of view, for example, during a motion through the entire workspace of the parallel mechanism, concepts are introduced to represent the parallel mechanism’s pose as a combination of *n* AprilTag poses.

The estimated poses of the AprilTags can be combined, for example, via weighted superposition. The required weights can be selected in different ways, for instance, by evaluating the optical distortion. In fact, objects in the optical center are less vulnerable to distortion than objects at the edges. Consequently, one could assume that AprilTags further away from the optical center provide less accurate poses than the ones in the optical center. The individual weights $w_{i}$ with i∈{1,…,n} can then be set depending on the distance to the optical center:

(11) $$\begin{array}{c} w_{i}=m\cdot \left({\left(\frac{cp_{x,i}}{c_{x,i}}-1\right)}^{2}+{\left(\frac{cp_{y,i}}{c_{y,i}}-1\right)}^{2}\right)+w_{\max}, \end{array}$$

where *m* is a selectable rate of change of weights, $\left[cp_{x,i},cp_{y,i}\right]$ are the coordinates of the *i*th detected AprilTag’s center points in pixels, $\left[c_{x},c_{y}\right]$ is the optical center, and $w_{\max}$ is the maximum weight value. Alternatively, the weights can be found by evaluating the quality of the AprilTags on the manufactured array. The accuracy of the estimated pose differs for each AprilTag. One could select weights empirically depending on the accuracy of every individual AprilTag. Thus, more accurate tags are weighted higher. The combined poses, here for the *x*-direction and the rotation angle α around the *x*-axis, are calculated as follows:

(12) $$\begin{array}{cc} x_{\mathrm{new}} & =\frac{w_{1}\cdot \left(x_{1}+△x_{1}\right)+w_{2}\cdot \left(x_{2}+△x_{2}\right)+\cdots +w_{n}\cdot \left(x_{n}+△x_{n}\right)}{w_{1}+w_{2}+\cdots +w_{n}}\;, \end{array}$$

(13) $$\begin{array}{cc} \alpha_{\mathrm{new}} & =\frac{w_{1}\cdot \alpha_{1}+w_{2}\cdot \alpha_{2}+\cdots +w_{n}\cdot \alpha_{n}}{w_{1}+w_{2}+\cdots +w_{n}}\;, \end{array}$$

where $△x_{i}$ is the distance in *x*-direction of the *i*th AprilTag to the center of the array. Note that this offset must be taken into account every time when calculating the translation, so that all AprilTags have a common reference point.

As an alternative to combine the AprilTag poses, averaging corrected for outliers can be used as $n_{x}\times n_{y}$ AprilTags in the field of view result in $n_{x}\times n_{y}$ measured values per image for the translations and rotations of the respective axes. In the first step, outliers are removed using median absolute deviation (MAD). Subsequently, the mean value of the remaining measured values is formed, resulting in more robust and less noisy measurements.

Last, but not least, instead of manually setting or calculating the weights, one can use neural networks. These can find the perfect weights which reduce the overall position error. Here, however, extensive training with reference data is required and the generalizability to other parallel mechanisms is not guaranteed.

#### Application Example: Data Fusion

For the hexapod, at least two times two AprilTags are visible in the camera’s field of view. In order to combine the poses computed from these AprilTags, averaging corrected for outliers is applied. In addition, the actual poses of the manufactured AprilTags on the AprilTag array were evaluated and the stored poses in the look-up table were updated.

## 5. Application

In the previous section, the proposed high-precision pose measurement system was designed. Now, it can be implemented in the state-of-the-art H-811.I2 6-axis miniature hexapod by Physik Instrumente (PI) GmbH & Co. KG. In the following, the experimental setup is revisited first. Thereafter, the calibration procedure is presented, which is required to precisely tune the camera matrix by linking the obtained image points with object points in real-world coordinates. This is followed by an accuracy analysis of our vision-based concept for absolute pose sensing. Among others, in the experiments, an investigation is performed where the minimum step sizes that can be resolved with a camera system in a single axis are analyzed. Furthermore, the system’s measurement accuracy is analyzed for multiple static poses in the hexapod’s workspace as well as for dynamic motions.

### 5.1. Experimental Setup

**Figure 16:**
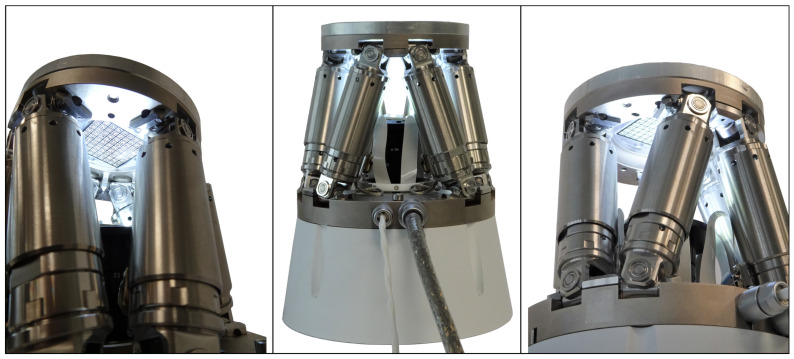
Photography of the investigated hexapod with the implemented vision-based concept for pose detection.

The proposed high-precision pose measurement system is implemented in the H-811.I2 6-axis miniature hexapod. As mentioned above, the hexapod has a translational travel range of ±17 mm, ±16 mm, and ±6.5 mm in *x*-, *y*-, and *z*-direction, respectively, a rotational travel range of ±10${}^{\circ }$ around the *x*- and *y*-axis, and a rotational travel range of ±21${}^{\circ }$ around the *z*-axis. An array of nine times nine AprilTags of the family 36h11 is mounted on the bottom side of the hexapod’s manipulator platform. The AprilTags have a size of 2.4 mm, are uniformly distributed on the array with a space of 1.3 mm, and are laser-engraved on a black laser foil. A camera with 5 MP resolution and a pixel size of 3.45 µm (MER2-503-36U3M by Daheng Imaging) and a ultra-high-resolution lens with a focal length of 35 mm is used (LCM-10MP-35MM-F2.8-1.5-ND1). An aperture level of f/8 and an exposure time of 3 ms is used. A ring light with 1.4 W power is mounted on the objective lens. For homogenous lighting conditions, the hexapod is completely covered. In order to reduce the minimum object distance, a 10 mm spacer is used. The spacer furthermore decreases the camera’s field of view. Consequently, the distance between the camera and the AprilTag array is 48 mm for the hexapod at z=0 mm. The camera is mounted in a housing that can be attached below the base platform of the hexapod. For solving the PnP problem, the implemented AprilTag algorithm is used and the parameters QD and NT are set to two and ten, respectively. Figure 16 shows the hexapod with the implemented pose measurement system from different views. It can be seen that the camera is mounted inside a white support structure and the hexapod is mounted on top of it.

### 5.2. Camera Calibration

The purpose of camera calibration is to link the obtained image points with object points in real-world coordinates. This is performed by selecting an appropriate camera model, for example, the pinhole camera model, and finding the camera’s intrinsic and extrinsic parameters. Intrinsic parameters are the camera’s focal length, skew, distortion, and the image’s center on the camera sensor, whereas extrinsic parameters are the camera’s position and orientation in the world. Both parameters can be found using calibration. Conventional camera systems are calibrated by using a calibration pattern. Here, checkerboard or square grids are used most commonly due to their identical and repetitive structure, see, for example, [59,60,61,77,78]. For large working distances with a correspondingly large field of view, such patterns can be produced without problems using a normal printer. The general calibration procedure is presented in the following.

For calibration purposes, it is assumed that the points of the object plane are all on the same xy-plane, so z=0 holds. The pinhole camera model can therefore be written as

(14) $$\underset{:=Y}{\underset{︸}{\left[\begin{array}{c} u\\ v\\ 1 \end{array}\right]}}=\underset{:=K}{\underset{︸}{\left[\begin{array}{ccc} b_{x} & 0 & c_{x}\\ 0 & b_{y} & c_{y}\\ 0 & 0 & 1 \end{array}\right]}}\left[\begin{array}{ccc} r_{11} & r_{12} & t_{1}\\ r_{21} & r_{22} & t_{2}\\ r_{31} & r_{32} & t_{3} \end{array}\right]\underset{:=X}{\underset{︸}{\left[\begin{array}{c} X\\ Y\\ 1 \end{array}\right]}}=HX$$

where *u* and *v* are coordinates of the image points Y. *X* and *Y* are coordinates of the object points X. The intrinsic parameters, consisting of the coordinates of the optical center $\left[c_{x},c_{y}\right]$ and the image distance $\left[b_{x},b_{y}\right]$, are stored in the camera matrix K. Both the intrinsic and extrinsic parameters that consist of the camera’s position and rotation in space are stored in the homography matrix H. Since the coordinates of the image plane and the coordinates in the object plane are known, H can be estimated by using direct linear transformation and singular value decomposition and a set of images taken at different angles and distances. For description, see [77,78].

If, however, the camera system is designed for small working distances, more precisely manufactured calibration patterns are necessary. In this concept, the manufactured AprilTag arrays are used as they also have an identical and repetitive structure. Furthermore, they are precisely manufactured and their sizes and distances are well known. The AprilTag library can be used to calculate the corner points of the AprilTags in the image. For a good calibration, each image should be shifted and rotated around each axis. Due to the comparably small working distance, the calibration images are not optimal. Here, only 15 images with six detected AprilTags are used and the camera matrix results in

(15) $$\begin{array}{c} K=\left[\begin{array}{ccc} 13813,1861 & 0 & 1224,1337\\ 0 & 13929,2375 & 1009,7503\\ 0 & 0 & 1 \end{array}\right]\;. \end{array}$$

In order to improve the calibration results, the following method is used. Since the camera’s resolution is known, the parameters $c_{x}$ and $c_{y}$ of the camera matrix K can be determined.

(16) $$\begin{array}{ccc} c_{x}=\frac{RES_{x}}{2}=1224\;\mathrm{px}\;, & \; & c_{y}=\frac{RES_{y}}{2}=1024\;\mathrm{px}\;. \end{array}$$

**Figure 17:**
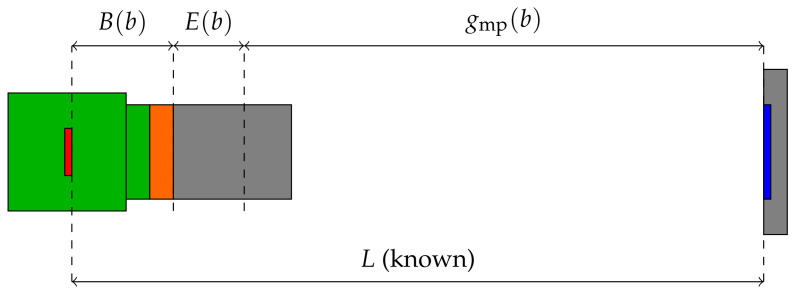
Schematic representation of the concept and the parameters used for calibration.

The image distances $b_{x}$ and $b_{y}$ correspond to the distance of the sensor to the optical main plane. They have the units mm/$px_{x}$ and mm/$px_{y}$, where $px_{x}$ and $px_{y}$ is the length of a pixel in *x*- and *y*-direction, respectively. As squared pixels can be assumed ($px_{x}=px_{y}=px$), this follows $b_{x}=b_{y}=b$. The AprilTag algorithm can be used to calculate the distance between the AprilTag array and the camera $g_{\mathrm{mp}}$ in *z*-direction. This calculated distance $g_{\mathrm{mp}}$ can now be compared with the actual distance between the AprilTag array and the camera. Knowing, for example, the total length *L* from the AprilTag to the camera sensor at a given position, the optimal image distance $b^{\prime }$ can be calculated by minimizing the error function E(b):

(17) $$\begin{array}{c} b^{\prime }=\underset{b}{arg min}\left|E\left(b\right)\right|=\min(|L-\left[g_{\mathrm{mp}}\left(b\right)+B\left(b\right)\right]\left|\right)\;, \end{array}$$

where B(b)=b·px is the image distance in meters and $g_{\mathrm{mp}}\left(b\right)$ is the distance between the AprilTag array and the camera calculated with the AprilTag algorithm for a given *b*. If the minimum is found, $g_{\mathrm{mp}}\left(b^{\prime }\right)+B\left(b^{\prime }\right)=L$ holds. Figure 17 illustrates the concept and the parameters used. For this method, the length *L* must be known very accurately. After calibration, the camera matrix K results in

(18) $$\begin{array}{c} K=\left[\begin{array}{ccc} 13742.116 & 0 & 1224\\ 0 & 13742.116 & 1024\\ 0 & 0 & 1 \end{array}\right]\;. \end{array}$$

With the known camera matrix K, the homography H and, thus, the parallel mechanism’s pose can be calculated; see, for example, [68].

### 5.3. Minimal Incremental Motion

In the first experiment, the minimal incremental motion that can be resolved with the proposed camera system is investigated. Therefore, different step sizes are tested around the focal plane (500 µm, 200 µm, 100 µm, 50 µm, 25 µm, and 10 µm, for all the axes, and 5 µm, 2 µm, 1 µm, 0.5 µm, and 0.25 µm in addition for the *x*- and *y*-axes). For each step size *S*, a staircase function with ten forward and ten backward steps is run along the investigated axis (while the other axes are held at zero) and at each position, 100 images are taken. The images are then fed to the PnP algorithm and the results are evaluated according the following criteria:

1. The norm of the average step size deviation $\left|\delta \right|$ shall not exceed 20% of the investigated step size *S*.
2. The standard deviation of the step sizes $S_{\mathrm{std}}$ shall not exceed 25% of the investigated step size *S*.
3. The maximum noise of the measured values $N_{\max}$ shall not exceed 25% of the investigated step size *S*.

If the criteria are fulfilled, the next-smaller step size is tested. These requirements are based on DIN ISO 230:2014-5 and ASME B5.54.2005 and are used in general in the qualification procedure of positioning axes, but they can also be used here since the same system parameters are considered, but instead of the positioning axis, the camera system is qualified. The parameters $\left|\delta \right|$, $S_{\mathrm{std}}$, and $N_{\max}$ can be calculated from the measurement data as follows:

(19) $$\begin{array}{cc} \delta & =\frac{S_{M}-S}{S}\;, \end{array}$$

(20) $$\begin{array}{cc} S_{\mathrm{std}} & =\sqrt{\frac{1}{2n-1}\sum_{i=1}^{2n}\left(\right|S_{i}{\left|-S_{M}\right)}^{2}}\;, \end{array}$$

(21) $$\begin{array}{cc} N_{\max} & =\max\left(\sqrt{\frac{1}{2n-1}\sum_{i=1}^{2n}{\left(x_{i}-{\bar{x}}_{i}\right)}^{2}}\right)\;, \end{array}$$

where $x_{i}$ is a set of 100 measurements taken for every position and ${\bar{x}}_{i}$ is the mean value of this set. The mean value of all the measured step sizes $S_{M}$ and the mean value of the *i*th measured step $S_{i}$ are calculated as follows:

(22) $$\begin{array}{cc} S_{M} & =\frac{1}{2n}\sum_{k=1}^{2n}\left|S_{i}\right|\;, \end{array}$$

(23) $$\begin{array}{cc} S_{i} & ={\bar{x}}_{i}-{\bar{x}}_{i-1}\;. \end{array}$$

As reference, poses obtained with the internal measurement system are used. This is possible as the hexapod itself has a minimal incremental motion that is probably a little better than the resolvable minimal incremental motion of the proposed sensor system (0.2 µm in *x*- and *y*-direction and 0.08 µm in *z*-direction, and 2.5 µrad around the *x*- and *y*-axis and 5 µrad around the *z*-axis). Furthermore, the implemented internal measurement system can resolve even smaller motions (few nanometers).

**Figure 18:**
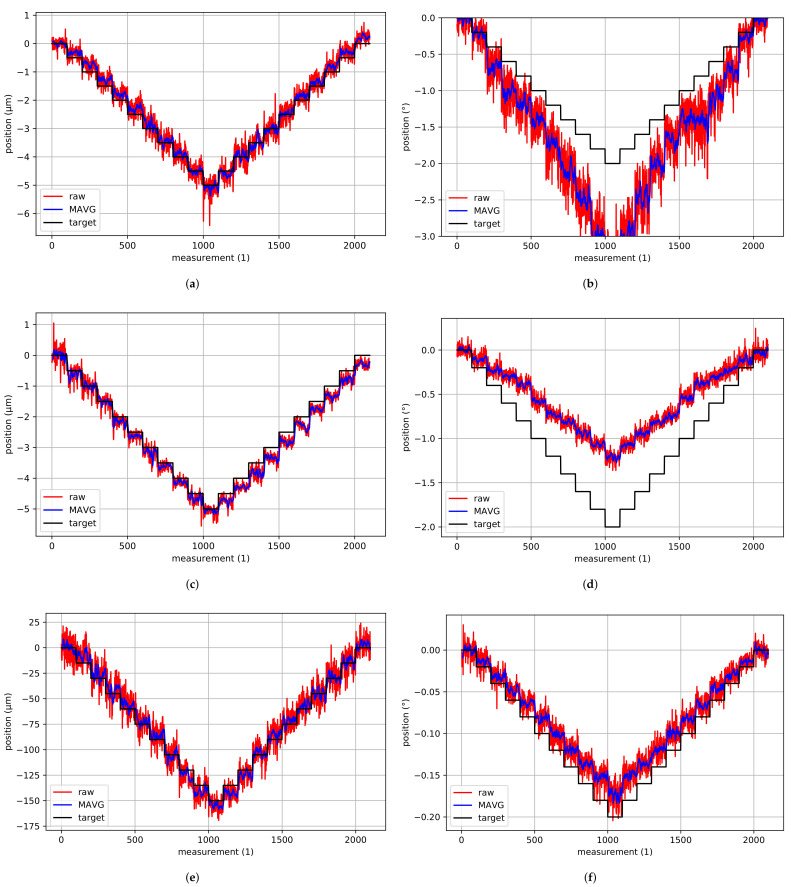
Experimental evaluation of the minimal incremental motion evaluation: Target (black), raw (red), and moving average (blue) values for given step sizes *S* obtained with the proposed direct and absolute vision-based pose measurement system: (**a**) Translation *x*-axis with S=0.5 µm, (**b**) Rotation *x*-axis with $S=0.2^{\circ }$, (**c**) Translation *y*-axis with S=0.5 µm, (**d**) Rotation *y*-axis with $S=0.2^{\circ }$, (**e**) Translation *z*-axis with S=15 µm, (**f**) Rotation *z*-axis with $S=0.02^{\circ }$.

Figure 18 shows representative results for the minimal incremental motion of the hexapod in every axis. Here, the target steps are shown in black and the raw measurement results obtained from the proposed direct and absolute pose measurement system are shown in red. Usually, the measurement data are passed through a low-pass filter. In contrast to that, here, a moving average with eight average data points is implemented. These results are shown in blue. Table 1 shows a summary of the minimal incremental motion and the obtained results for the criteria. The table furthermore shows a column where the maximum noise of the moving average values are evaluated.

From Figure 18 and Table 1, it can be observed that motions along the *x*- and *y*-axis can be observed very accurately. Here, a minimal incremental motion of 0.5 µm is possible. The same applies for the rotation around the *z*-axis. Here, a minimal incremental motion of $0.02^{\circ }$ (0.35 mrad) is possible. Motions along the *z*-axis can be resolved significantly worse, and only step sizes bigger than 15.0 µm fulfill the criteria. Rotations around the *x*- and *y*-axis can also be resolved comparably inaccurately. Here, only a minimal incremental motion of $0.2^{\circ }$ (3.49 mrad) is possible. This, however, is quite obvious as a rotation around the *x*- and *y*-axis of the hexapod basically affects a motion in *z*-direction of the AprilTags’ corner points.

From Figure 18 and Table 1, it can furthermore be noticed that measurement noise is the criterion that most likely failed. Using a moving average with eight average data points, obviously lower measurement noise can be obtained. However, ever lower measurement noise would be required to fulfill more advanced criteria. Last, but not least, it can also be noticed that, for motions along the *z*-axis and for rotations around the *x*- and *y*-axis, the detected step size does not fully match with target ones. In fact, the average step size deviation lies at 11.08% for the the *z*-axis (which corresponds to 1.66 µm) and 76.27% ($0.15^{\circ }$) and 39.88% ($0.07^{\circ }$) for rotations around the *x*- and *y*-axis. For the *z*-axis, this might result from errors in the calibration resulting in a smaller image distance *b* or manufacturing errors of the AprilTag resulting in a smaller AprilTag size. For rotations around the *x*- and *y*-axis, the step size deviation can be caused by an non-perfect choice of the hexapod’s pivot point. In fact, if the distance between the AprilTag array and the pivot point is not chosen correctly, rotations around the considered pivot point do not correspond to the measured rotations.

Reconsider Section 4.2, where the camera system was chosen and the smallest detectable unit ϵ was estimated. For no subpixeling (spx=1), an ϵ of 10.65 µm was obtained (1.065 µm for spx=0.1) for the chosen camera and objective lens. From Figure 18 and Table 1, it can be concluded that, especially for the *x*- and *y*-axis, even smaller units can be detected. Here, a subpixeling parameter spx of 0.05 can be assumed. For the *z*-axis, the resolvable step size of 15.0 µm does not correspond to the smallest detectable unit ϵ as our estimation in Section 4.2 is only valid for measurements within the sensor plane, which is not the case for the *z*-axis.

### 5.4. Static Poses in the Hexapod’s Workspace

In a second experiment, the measurement accuracy is evaluated for five randomly chosen static poses in the hexapod’s workspace. For every pose, 500 images are acquired and the pose for the AprilTag in the center of the AprilTag array is evaluated. The measured translations *x*, *y*, *z* and rotations α, β, γ are averaged and compared with the values of the internal measuring system of the hexapod. Table 2 shows the root mean squares (rms) and the standard deviations (std) of the position and orientation errors Δ for every pose.

Similarly to the results for the resolvable minimal incremental motion, the results for the *x*- and *y*-components, as well as for the rotation around the *z*-axis, are better than the others. The *z*-components especially show significantly higher standard deviations. In fact, the errors’ standard deviations for the *z*-axis range from 10.38 µm to 73.20 µm and therewith are 10 to 100 times higher than for the *x*-axis and two to 60 times higher than for the *y*-axis. For the errors’ root mean squares, comparably similar values are obtained for the axes that range from 2.91 µm to 218.48 µm. For the rotation angles α, β, and γ, rotations around the *z*-axis have the least rms and std values. For the rotations around the other axes, higher errors were obtained, where the errors’ standard deviations are four to 30 times higher for α and nine to 20 times higher for β, compared to rotations around the *z*-axis γ. The same applies for the errors’ root mean squares.

### 5.5. Dynamic Motion Accuracy

In a third experiment, it is investigated how accurate a dynamic motion of the H-811.I2 6-axis miniature hexapod can be resolved. As reference, the poses obtained with the internal measurement system are used. First, a 1 mm step in the *x*-direction is performed by the hexapod, and the poses obtained with the internal measurement system and the proposed high-precision pose measurement system are compared. The hexapod moves with a maximum velocity of 10 mm/s and with a maximum acceleration of 11.6 mm/s${}^{2}$. Afterwards, the same is performed with a 0.5 mm step in the *z*-direction. For the proposed vision-based pose measurement system, 20 measurements per second was achieved while the internal measurement system was set to 1000 measurements per second.

**Figure 19:**
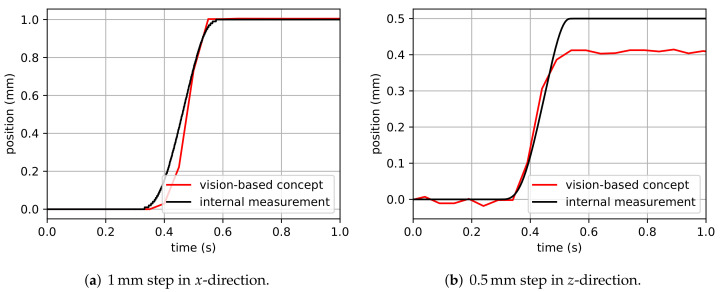
Measured positions for a dynamic motion of the hexapod (**a**) in *x*-direction and (**b**) in *z*-direction obtained with the proposed vision-based pose measurement system (in red) and the internal measurement system (in black).

Figure 19 shows the measured positions obtained with the proposed vision-based pose measurement system and the internal measurement system. In general, it can be noticed that the vision-based pose measurement system can follow the motion of the hexapod very well. Especially for the motion in *x*-direction, the results of the vision-based measurement system match the results of the internal measurement system very accurately. A small delay can be recognized before the motion is detected by our proposed vision-based measurement system. Nevertheless, for the tested 1 mm step, a step size of 1.0046 mm was obtained, a deviation of 0.4605%. For the motion in *z*-direction, however, the results obtained with our vision-based measurement system are comparably noisy and, more importantly, the desired step size of 0.5 mm was not detected. In fact, a step size of 0.4077 mm was obtained, a deviation of −18.46%, which is similar to the step size deviations obtained in Section 5.3. Note that the obtained deviations are taken with respect to the internal measurement system that estimates the hexapod’s pose from the linear actuators’ lengths. It is possible that, although the lengths correspond to the desired motion, the hexapod’s manipulator platform moves differently, caused, for example, by backlash or temperature deformation.

### 5.6. Measuring Temperature Drifts in the Hexapod

**Figure 20:**
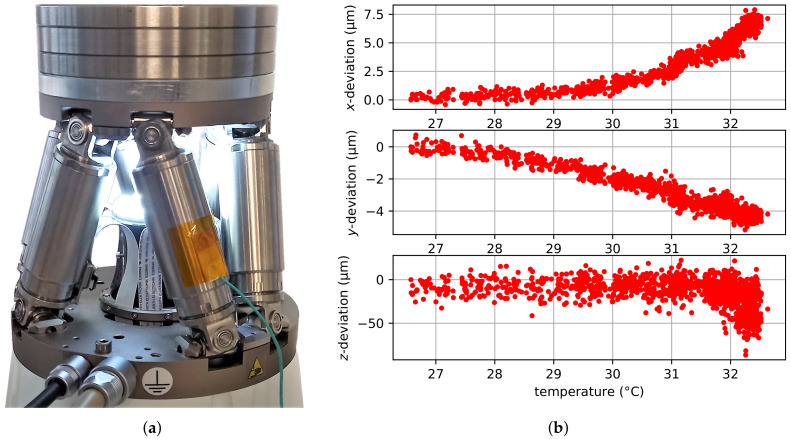
Unwanted motion of the hexapod’s manipulator platform due to temperature deformations of the linear actuators measured with the proposed vision-based pose measurement system and the internal measurement system: (**a**) experimental setup and (**b**) measured deviations in the *x*-, *y*-, and *z*-direction independent.

In Section 1, the limitations of indirect pose measurements were mentioned. These include backlash and temperature deformations in the active joints that cannot be detected from the internal sensor system. In order to prove that the proposed vision-based pose measurement system is capable of measuring these unwanted motions, an additional experiment is performed, where the temperature drift in the hexapod is measured. This is performed by moving the hexapod to a designated pose and keeping it there. Here, the hexapod’s home position is chosen as target pose. Furthermore, an additional weight of 1.5 kg is mounted on the hexapod’s manipulator platform to increase the self-heating process; see Figure 20a. Consequently, the hexapod requires energy to keep the pose and will slowly heat up, which will result in unwanted temperature deformations of the linear actuators. The internal measurement system, however, cannot see this motion. In contrast to that, the proposed vision-based pose measurement system is capable of measuring these unwanted motions of the hexapod’s manipulator platform. The poses measured over a two-hour period with ten measurements per minute are shown in Figure 20. They are plotted over the temperature measured at the linear actuators of the hexapod via a PT100 temperature sensor.

Within this two-hour period, a temperature increase of 7 ${}^{\circ }$C was measured. The measurement results show that there exists a thermal drift in the hexapod. The thermal drift for the *x*- and the *y*-axis is nearly constant and lies at 1 µm/${}^{\circ }$C and –0.6 µm/${}^{\circ }$C, respectively. In the *z*-direction, no real thermal drift was detected. Here, however, the measured values spread over a wide range of 20–30 µm. For temperatures above 32 ${}^{\circ }$C, it seems that the measured values start to drift towards −50 µm. It thus seems that the temperature in the linear actuators affects the pose of the hexapod’s manipulator platform and, as the pose is not directly measured, temperature drifts cannot be controlled.

As an alternative direct measurement method to detect the temperature drift, an interferometer can be used. This, however, would require a more complex measurement setup and it is only possible to measure the drift in one direction (along one axis). A combination of multiple interferometers are considerable, but their perpendicular alignment increases the complexity of the measurement setup. Last, but not least, a coordinate measurement machine is an alternative. However, the costs, setup, and the required measurement time usually do not pay off for such application.

## 6. Summary, Discussion, and Future Work

In this paper, a new vision-based sensor concept for parallel mechanisms was presented. By implementing a camera module on the base platform and an array of fiducial tags on the moveable manipulator platform of a parallel mechanism, a highly accurate, direct, and absolute pose measurement system can be obtained. The proposed measurement system can thus overcome the limitations of indirect pose measurements in parallel mechanisms where, usually, the active joints’ coordinates are measured to solve the direct kinematics problem and thus find the manipulator platform’s pose. The known concepts are limited by the nature of indirect measurements. A deformation of the manipulator platform, for example, cannot be measured with the internal measurement system as it has no influence on the linear actuators’ lengths measured via encoders. In contrast, the proposed vision-based sensor concept is able to detect this deformation as well as pose errors resulting from backlash and temperature drift, as it directly measures the manipulator platform’s pose.

### 6.1. Summary

In this paper, the design of such a vision-based pose measurement system and external and internal restrictions, as well as selectable parameters, were investigated step by step and applied to the example of a state-of-the-art hexapod, the H-811.I2 6-axis miniature hexapod by Physik Instrumente (PI) GmbH & Co. KG. In the last part of this paper, the proposed high-precision pose measurement system was finally applied on the H-811.I2 6-axis miniature hexapod and several investigations of the minimum resolvable step size, as well as the measurement accuracy, were performed.

In the experiments, it was proven that the proposed vision-based pose measurement system is capable of measuring poses with a micrometer, and even nanometer, accuracy. The translational accuracy analysis showed that step sizes of 0.5 µm in *x*- and *y*- direction and 15.0 µm in *z*-direction are detectable with a 5 MP camera, an objective lens with a focal length of 35 mm, and a 10 mm spacer. For the rotational accuracy, step sizes of $0.02^{\circ }$ for the *z*-axis and $0.2^{\circ }$ for the *x*- and *y*-axis were resolvable with high accuracy. By reducing the measurement noise, for example, by using a moving average, even higher accuracies can be obtained. A comparison of different PnP algorithms showed that pose detections via the AprilTag algorithm provide the best results in terms of accuracy and robustness. The achievable sampling rate ranging from 3.5 to 30 measurements per second mainly depends on the parameters QD (quad decimate), NT (number of threads), and the number of AprilTags in the camera’s field of view.

The performed investigations furthermore show that the depth of field has a huge impact on the detection rate. The further away the AprilTag array is positioned from the camera’s focal plane, the lower the detection rate. Here, a compromise must be found between the camera’s depth of field and the workspace of the parallel mechanism. Homogeneous illumination is furthermore crucial for obtaining optimal image data. Uneven exposure can cause the detected corner points to fluctuate, leading to scatterings in the measurement results. In the case of overexposure with incident light, reflections on the white border may cause the AprilTags to be overlit from the outside. This reduces the distance between the detected corner points and leads to errors in pose estimation. For dynamic measurements, a camera with global shutter should be used, as rolling shutters cause motion blur and distortion.

Due to the absolute pose measurement, the proposed vision-based sensor concept can also observe undesired motions in parallel mechanisms. In the experiments, for example, it was shown that the proposed vision-based pose measurement system can measure temperature deformations in the H-811.I2 6-axis miniature hexapod that cannot be measured from the internal measurement system. As mentioned above, it can detect even more of these undesired motions. One example is backlash in the passive joints as well as in the linear actuators. The vision-based sensor concept is, furthermore, able to measure deformations or drift due to mechanical stress and is thus capable of determining the mechanism’s stiffness. In addition, weird errors that can occur in the linear actuators can also be measured. As an example, consider a soiled encoder that misses some steps. Consequently, the linear actuator’s length does not correspond to the required length which, however, is not detected by the internal measurement system and is thus not corrected. In consequence, the hexapod will slowly drift away. By using our proposed vision-based pose measurement system, such an error can be measured and corrected.

The proposed vision-based pose measurement system can be considered as an alternative to expensive coordinate measuring machines. A coordinate measuring machine measures the geometries of physical objects by using tactile and non-tactile probes to detect discrete points on the objects’ surfaces. Usually, coordinate measuring machines are very expensive, can measure with high velocities and accelerations, and have a huge working range resulting, however, in a comparably low accuracy (micrometer range) that furthermore depends on the measured length.

### 6.2. Discussion and Future Work

Using the internal measurement system of the H-811.I2 6-axis miniature hexapod as a reference to compare the accuracy of our proposed vision-based pose measurement system is unusual, as the limitation of indirect pose measurements in parallel mechanisms was discussed in Section 1 of the paper. However, the internal measurement system of the H-811.I2 6-axis miniature hexapod can resolve incremental motions of 5 nm with a high measurement rate of up to 10,000 measurements per second, and is thus capable as a reference for the accuracy of relative motions. Nevertheless, for estimating the absolute accuracy and, finally, for qualifying our vision-based pose measurement system, a direct absolute pose measurement mechanism such as a coordinate measuring machine is necessary.

The calibration procedure that was presented in this work requires very accurate knowledge of the length *L*, which is the length between the camera sensor and the AprilTag array. For repeatability, a highly-accurate gauge can be manufactured (whose length is known) that can be placed between the camera, e.g., the front of the camera, and the AprilTag array. Here, the distance between the camera sensor and the front of the camera is required; a value that is usually provided by the manufacturer.

The obtained accuracy of our proposed vision-based pose measurement system can be further improved by several adoptions. Bigger AprilTag sizes can be used, resulting in a higher resolution. However, less AprilTags could be seen in the camera’s field of view. By using transmitted light illumination instead of direct incident lighting, a higher contrast and smoother edges can be achieved. The higher light intensity additionally reduces the influence of stray light as well as the required exposure time of the camera. Instead of determining the reference points in the image from corner points of squared fiducial tags, circles or ellipses can be used, as mentioned in [54]. In case of overexposure, the detected center points of circles or ellipses change insignificantly compared to corner points and less noisy measurement values can be obtained.

Furthermore, it is possible to replace the passive AprilTag array with an active array, for example, an LCD display or an electrophoretic display. In this way, possible errors from the fabrication of the array can be avoided. The accuracy of the applied fiducial tags then depends on the resolution and quality of the display. To further increase the achievable accuracy in the *z*-direction, either multiple cameras, e.g., stereo camera systems, or RGB-D cameras can be used; see, for example, [79]. RGB-D cameras have an infrared sensor in addition to the RGB color sensor to obtain depth information. However, since the depth information obtained is a point cloud, a much higher computational effort is required.

The proposed concept for a vision-based pose detection has several application possibilities in addition to the above-presented standalone measurement system. It can also be used, for example, for calibration purposes. Here, the combined error of actuator backlash, joint backlash, and temperature drift can be detected and stored in the mechanism’s controller. Thus, the faulty pose can be corrected and a higher absolute accuracy can be achieved. Since calibration tasks are not run-time critical, measurements can be performed over a longer period of time and computationally intensive filters can be used. Therefore, a higher accuracy can be achieved as only the raw measurement values were used for the evaluations in this work. Provided that the sampling rate is high enough, the concept can also be integrated as a measuring system in a closed-loop control in addition to an internal measurement system. The errors mentioned can then be compensated directly by the controller. In addition to the above-mentioned applications, the proposed vision-based pose measurement system can be used to reduce product development times. This practical advantage is possible as the proposed sensor concept can be used for precise measurement tasks such as measuring the guidance accuracy, linearity, or repeatability. Furthermore, it can be installed comparably easy on any mechanism so that complex and expensive setups such as interferometers or coordinate measuring machines can be avoided. It hereby represents a combination of simple design with nevertheless precise direct measurement. It can, therefore, be used especially in rapid-prototyping processes. Last, but not least, the application range is not limited to parallel mechanisms and can be extended to linear stages, serial robots, or magnetic levitation platforms. The proposed vision-based pose measurement system is ideal especially for planar stages, as the motion in *z*-direction is zero, so a camera system with a high amplification and, thus, a very small depth of field can be used, resulting in a high position accuracy.

## 7. Patents

Parts of this work were patented in the following patent application: Schulz, S.; Schempp, C.: Posenbestimmung bei Parallelkinematiken mit Referenzmarkern. 25 Nov. 2021, German Patent Application No. 10 2021 213 358.4.

## Figures and Tables

**Table 1:** Minimal incremental motion *S* for every axis and the obtained results for the following criteria: The norm of the average step size deviation |δ|, the standard deviation of the step sizes $4\cdot S_{\mathrm{std}}$, and the maximum noise of the measured values $4\cdot N_{\max}$.

Motion	Axis	*S*	|δ|	$4\cdot S_{\mathrm{std}}$	$4\cdot N_{\max}$	${\mathrm{MAVG}}^{\left(8\right)}$
translation	*x*	0.5 µm	3.67%	0.38 µm	1.93 µm	1.18 µm
translation	*y*	0.5 µm	1.28%	0.41 µm	1.3 µm	0.81 µm
translation	*z*	15.0 µm	11.08%	7.01 µm	47.38 µm	19.06 µm
rotation	*x*	0.20${}^{\circ }$	76.27%	0.50${}^{\circ }$	1.27${}^{\circ }$	0.49${}^{\circ }$
rotation	*y*	0.20${}^{\circ }$	39.88%	0.15${}^{\circ }$	0.29${}^{\circ }$	0.13${}^{\circ }$
rotation	*z*	0.02${}^{\circ }$	0.29%	0.0094${}^{\circ }$	0.058${}^{\circ }$	0.021${}^{\circ }$

**Table 2:** Investigated static poses in the workspace of the hexapod and the corresponding root mean squares (rms) and standard deviations (std) of the position and orientation errors Δ for every pose.

Pose	rms	std	rms	std	rms	std	rms	std	rms	std	rms	std
[*x*, *y*, *z*, α, β, γ]	Δx (µm)		Δy (µm)		Δz (µm)		Δα (${}^{\circ }$)		Δβ (${}^{\circ }$)		Δγ (${}^{\circ }$)	
[−1.64, −3.12, −0.39, −1.78, −4.78, 7.57]	70.85	0.38	55.30	0.42	322.41	10.38	0.31	0.16	3.91	0.15	0.16	0.02
[−0.53, 1.51, 0.79, 5.15, −2.74, −4.66]	2.91	0.68	136.34	1.11	171.61	65.56	4.69	0.43	1.51	0.47	0.14	0.02
[−1.33, 1.66, −2.90, 2.87, 3.21, 0.10]	9.44	1.70	142.75	1.35	80.84	73.20	1.97	0.48	2.43	0.29	0.08	0.01
[0.15, 4.58, 0.30, −5.02 4.72, 6.95]	21.22	3.19	34.47	15.11	164.51	36.97	1.23	0.64	3.94	1.79	0.17	0.13
[−4.77, 1.44, −0.30, −3.46, −4.91, −7.36]	102.81	2.36	124.54	0.81	218.48	31.73	2.75	0.20	6.15	0.26	0.46	0.02

**Figure A1:**
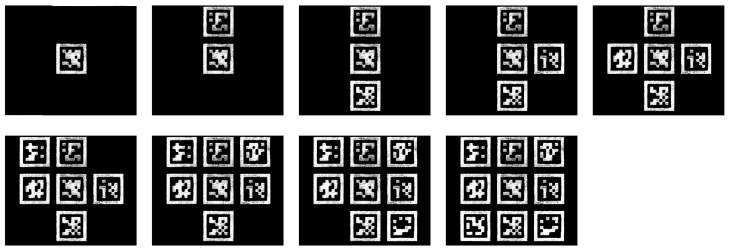
Images that are used for investigating the influences of QD (quad decimate), NT (number of threads), and the number of AprilTags in the field of view on the execution time.

## Data Availability

The data that support the findings of this study are available from the corresponding author upon reasonable request.

## Appendix A

In this supplementary section, a detailed description of our experiments in Section 4 is given. All experiments were performed on the H-811.I2 6-axis miniature hexapod by Physik Instrumente (PI) GmbH & Co. KG shown in Figure 1.

### Appendix A.1. Experiments in Influence of the Working Distance

To measure the influence of the working distance on the detection rate, for each spacer, the AprilTag array is positioned in the focal plane. From here, the AprilTag array is moved in increments of 0.1 mm towards ($+T_{z}$) and away ($-T_{z}$) from the camera. Images are taken and the detection rate is calculated relative to the number of tags that are fully visible at each position; see Figure 9a–d. A detection rate of 100% indicates that all the tags in the camera’s field of view are detected. This area is defined as the focal area. Lower detection rates imply that one or more tags in the camera’s field of view are not detected correctly. This indicates the limit of the camera’s depth of field. The border area is the area where AprilTags are still detected but the image is already blurry (indicated by a detection rate of less then 100%). By increasing the working distance starting from the focal area, the detection rate decreases in the border area and, in the end, abruptly reaches 0%. From the results, it can be noticed that the spacer size has a major impact on the accessible working distance.

To demonstrate the different accuracies of the detected poses in the focal and the border area, a staircase motion in the *z*-direction with a step size of 0.6 mm is driven with the hexapod. The staircase motion consists of ten forward and ten backward steps. For every step, 100 images are taken (resulting in 2100 measurements) and the pose is calculated with the PnP algorithm.

In the application part of this section, the influence of a spacer on the pose accuracy is investigated. A staircase motion in *z*-direction with a step size of 15 µm is performed with the hexapod. For every step, 100 images are taken and the pose is calculated with the PnP algorithm. This experiment is performed multiple times for different setups: It is performed (a) without spacer in the focal area, (b) with a 10 mm spacer in the focal area, and (c) with a 10 mm spacer in the border area.

### Appendix A.2. Experiments in Illumination

To investigate the influence of illumination on the detected distance in *z*-direction, the hexapod is covered with a box to measure under homogenous lighting conditions. The hexapod pose is held at a constant position and the distance is calculated via PnP algorithm. As constant position, the hexapod’s home position is used. The exposure time and the aperture stop are varied systematically to investigate their influence on the measured *z*-direction. For each measurement setup, 60 images were taken.

### Appendix A.3. Experiments in PnP Algorithms

In the application part of this section, the influence of the PnP algorithm on the pose accuracy is investigated. Therefore, a staircase motion in *x*-direction with a step size of 0.5 µm is performed with the hexapod, and for every step, 100 images were taken. The AprilTags’ corner points were extracted in pixel coordinates and passed to the presented algorithms with the corresponding object points that were stored in a look-up table. From this, the hexapod’s pose is calculated.

### Appendix A.4. Experiments in Data Handling

The AprilTag algorithm that suits best for calculating the hexapod’s pose from the AprilTags’ corner points has several parameters. Among others, these are the QD (quad decimate), NT (number of threads), and the number of AprilTags in the field of view. To investigate the influences of these parameters on the sampling rate, they are systematically varied. Here, only one picture is used for every experiment. Originally, nine AprilTags are visible in the image. To reduce the number of visible AprilTags, image editing software was used to remove the desired number of AprilTags. The images are shown in Figure A1.
